# High-school adolescents’ motivation to rugby participation and selection criteria for inclusion in school rugby teams: coaches’ perspective (the SCRuM project)

**DOI:** 10.1186/s13104-019-4138-y

**Published:** 2019-02-26

**Authors:** M. Chiwaridzo, G. Ferguson, B. C. M. Smits-Engelsman

**Affiliations:** Division of Physiotherapy, Department of Health and Rehabilitation Sciences, Faculty of Health Sciences, University of Cape Town, Observatory, Cape Town, South Africa

**Keywords:** Adolescents, Motivation, Qualitative study, Coaches, Rugby, Zimbabwe, SCRuM

## Abstract

**Objective:**

Despite increasing rugby popularity among schoolboys’ worldwide, specific factors influencing their motivation to participate in rugby remain unclear. Therefore, this study was conducted in two parts with a dual purpose of exploring perceptions of rugby coaches on (i) factors motivating schoolboys to engage in competitive rugby, and (ii) criteria for selecting schoolboy rugby players for possible inclusion in school rugby teams.

**Results:**

A qualitative study targeting Zimbabwean high school-based rugby coaches purposively-recruited during the 2017 Dairiboard Zimbabwe Rugby School Festival was conducted. Using the conventional approach to content analysis, the 22 recruited male coaches (median age = 45.5 years) felt that playing rugby is a choice largely influenced by either intrinsic or extrinsic motives for schoolboys. Additionally, coaches considered players’ characteristics (performance during training, attitude, physical qualities and skills) and match-related factors when selecting schoolboys for possible inclusion in school rugby teams. To effectively promote competitive rugby participation among schoolboys and promote sustainable and effective talent identification programmes in Zimbabwe, more recognition should be paid to factors motivating schoolboys to participate in rugby and also on the factors coaches consider when assembling school rugby teams which indirectly informs on what coaches think should be trained among schoolboy rugby players.

**Electronic supplementary material:**

The online version of this article (10.1186/s13104-019-4138-y) contains supplementary material, which is available to authorized users.

## Introduction

Rugby union (rugby) is a popular sport among schoolboys worldwide [1–3]. Today, schoolboy rugby players are playing more competitive matches and have better physical attributes than before [4–6]. Studies points to increased physical demands of schoolboy rugby and high injury risk [7–10]. Still, schoolboy rugby continues to grow worldwide [1]. In Zimbabwe, the growth is evidenced by establishment of the “elite” Super Eight Schools Rugby League (SESRL) and the “sub-elite” Co-educational Schools Rugby League (CESRL) [4, 11]. However, little is known about factors influencing motivation to participate in competitive rugby from the coaches’ perspective.

Several studies documented general reasons for sports participation for children, adolescents and adults ranging from weight management, social interaction to enjoyment [12–18]. Innumerable reasons cited illustrate complexity of the construct of motivation [18, 19]. One motivational theory, the self-determination theory (SDT) widely used to explain participatory behaviour [19, 20], is conceptually anchored on the belief that motivation is either intrinsic or extrinsic-oriented and driven by psychological needs for competence, autonomy and relatedness [21, 22].

Important to understand also is whether players’ attitudinal attributes could be a selection strategy coaches use when assembling a school rugby team. Studies conducted to investigate rugby coaches’ selection criteria of schoolboys are scarce. Previous studies showed that team selection is based on players’ experience, and certain attributes [23–25]. However, this evidence is mainly from quantitative studies. Therefore, this study was conducted to explore perceptions of rugby coaches on reasons they believe motivate schoolboys to participate in rugby and strategies used for selection of school teams. This study is useful in Zimbabwe where schoolboy rugby is considered an “elite” sport popular in private and top government schools, and where inclusivity efforts to develop the sport in disadvantaged schools are underway. Practically, this study may inform development of strategies to increase participation rates among schoolboys. Moreover, results on selection criteria may inform coaches on qualities important for rugby from the coaches’ perspective.

## Main text

### Research design, setting and participants

A qualitative study was employed for this study. This study was part of a broader project called the School Clinical Rugby Measure (SCRuM) partly described elsewhere [11]. Data collection was conducted at a school hosting the 2017 Dairiboard Zimbabwe Schools Rugby Festival (DZSRF). The festival was a 7-day long tournament featuring 150 high-schools. Coaches were selected with an *apriori* intention of maximum heterogeneity within the sample. This entailed purposively recruiting coaches from three high-school rugby leagues in Zimbabwe, namely SESRL, CESRL and Interscholastic High Schools Rugby League (IHSRL, “amateur league”) [11]. Full-time rugby coaches with at least 5 years’ coaching experience were eligible.

### Procedure

Ethical approval was granted by the University of Cape Town Human Research Ethics Committee (HREC ref: 016/2016). Coaches were approached by the first author, and interview appointments set with willing coaches. Written informed consents were provided. Subsequently, a trained research assistant conducted semi-structured, one-on-one interviews in a quiet classroom using an English interview guide [26]. All interviews were audio-recorded and lasted between 15 and 40 min. The recordings were then transcribed verbatim. Participant checking of the transcripts was conducted with a convenience sample of participants (n = 12) checking accuracy of the transcripts.

### Data analysis

A conventional inductive approach to content analysis [27] following steps of “decontextualisation”, “recontextualisation” and “categorisation” as described by Bengtsson [28] was used for theme generation. This entailed reading the transcripts, extracting and condensing meaning units, data coding, formulating categories and themes. This approach allowed themes to flow from the data [27] and was chosen because of limited literature explaining schoolboy participation in rugby and coaches’ selection criteria. Data analysis was manually completed by the first author and subsequently triangulated by an independent person.

### Results

#### Sample characteristics

The coaches’ demographic and work-related data are presented elsewhere [26]. Briefly, 22 coaches were interviewed. The majority had ≥ 10 years coaching experience and were mainly coaching senior schoolboys from private schools playing in the SESRL.

#### Motivation for the participatory behaviour

Coaches’ perceptions on factors motivating schoolboys’ participation in rugby yielded one over-arching theme (“*It is a choice to play rugby*”). Schoolboys are motivated by intrinsic (personal preference, enjoyment, and nature of the sport) or extrinsic factors (influence from significant others, professional ambitions, emulation and monetary rewards) (Additional file 1).

##### Intrinsic factors

Most coaches felt that rugby is a personal preference.

*“The sport is open to every child […]. Any child who wants to play, we encourage them to join in and play. […]. Our own Ministry of Primary and Secondary Education […] mandates every child to participate in sport….” (Participant AW01).*

*“You know rugby is a sport for everyone, not just for the 30 players you see running around for the ball in the pitch…” (Participant STG01)*

Some indicated that schoolboys are motivated by enjoyment and passion.

*“I think what motivates these youngsters to play rugby is passion for the game, the kids enjoy the sport […]. If you consider how dangerous the sport […] and still you have 30, 40 kids coming and say they want to play rugby. That is passion.” (Participant CBC01).*

*“I remember asking one Under*-*16 rugby player, after he walked out of a competitive match last year, why he is still interested in playing rugby after having a fracture of collarbone. He said to me rugby is everything to me; it’s all I feed on every day. I read, sleep and talk rugby….” (Participant MF01).*

Schoolboys were also said to be motivated by the nature of the sport (physicality, accommodativeness and spectator magnetism).

*“….watch when my boys play School A, it’s like war, [….], and most students who come for rugby […], probably enjoy the adrenaline rush that comes with tough and competitive sports…” (Participant AW01).*

*“….which sport can accommodate players of different sizes of people like rugby. You see very fat kids play rugby, very thin kids playing rugby and in*-*between players playing rugby….” (Participant LOM01).*

*“….so that excitement of playing competitive rugby, in a sport that attracts a huge crowd at the school is probably overwhelming [….] and makes them want to play rugby for the school.” (Participant F01).*

##### Extrinsic factors

Professional ambitions, emulation and monetary rewards were also mentioned.

*“….most of these boys […], play rugby with the hope […] that they will play professional rugby […] and probably make a lot of money. That mentality drives their passion […], and they feel motivated to want to play rugby.” (Participant MF01)*

*“….we have the likes of coach X, who came through the junior ranks at this school […], played outside Zimbabwe and now they are back coaching these kids. That is strong motivation for the kids….” (Participant PE01)*

External influences were also cited.

*“…some are dragged by others, especially if you look at the U13s…..” (Participant ES01).*

*“There are also other influences […] from parents who used to play the sport when they were young, or used to enjoy watching the sport….” (Participant AW01).*

#### Selection criteria

Analysis of the factors coaches consider for selection of schoolboys into teams yielded two overarching themes (Additional file 2).

##### Theme 1: “It is about the player”

Most coaches consider players performances during training.

“*The youngsters that have been brilliant in training, working very hard during training as seen by the coach probably get a nod to play.” (Participant PE02).*

Furthermore, attitudinal traits such as motivation were highlighted.

*“I choose players that are hard*-*working […], and rugby is a principled sport, so we expect the boys to have principles, to show that they are committed, motivated to play and have discipline…” (Participant ER01)*

Some considered fitness, physical qualities and rugby skills.

*“….. But of course they have to have the physical qualities and the skills that important in rugby, and they have to be injury*-*free.” (Participant P01).*

*“The sport is for the strong [….], there is a degree of physical fitness that we expect all rugby players to have, minimum physical fitness […]” (Participant MF01).*

*“…but certainly we would pick players who can run, who can hold the ball, pass and score [….]players who are strong to tackle, [….], players who can fight to win possession of the ball….” (Participant ES02).*

##### Theme 2: “It’s about the match”

Some coaches felt that team selection is circumstantial, factoring in the impending competitive match

“*I try to go for players whom I know will give me a win, a balanced team […]. So the team must be balanced….” (Participant ER02).*

*“……So as a coach, you need to have good knowledge of your opponents, their strength and weakness and form strategise and see which players from your team you need to include in the team.” (Participant STG01).*

### Discussion

This study explored factors coaches believe motivate schoolboys’ participation in competitive rugby and strategies to select them for school rugby teams. Unlike most studies [15, 18, 19], this study uniquely sought coaches’ perceptions. Coaches represent an important stakeholder in continued rugby participation by schoolboys. Additionally, by virtue of their experience and multifaceted roles as recruiters, trainers, and team selectors, coaches have keen insight into schoolboys’ motivation. They offer unique perspectives which may guide the development and implementation of strategies to improve schoolboys’ participation rates.

The main finding of this study shows that coaches have different reasons explaining participation in rugby. Motives range from personal preference, enjoyment, passion, nature of the sport, professional ambitions, influence from parents and friends. This diversity highlights complexity of the construct of motivation [29]. Our results also seem to imply that schoolboys’ participation is an individualistic decision underpinned by intrinsic or extrinsic factors and aimed at realising certain goals. This is the foundational basis of the SDT [18, 21, 22, 30]. The theory assumes that human behaviour is driven by psychological needs of “competence”, “autonomy” and “relatedness” [21]. Our results support the conceptual principles underpinning SDT. For example, the need for autonomy is evidenced by the independent decision making to engage in competitive rugby. Additionally, the need for relatedness as described in literature [22] helps us understand the influence of significant others in motivating schoolboys.

Although there are no studies documenting coaches’ perceptions on factors motivating schoolboys to participate in rugby, our results are consistent with studies investigating general sports participation among children, adolescents and adults [12, 14, 15, 17, 18]. A study conducted using college students’ found that enjoyment and the need for challenges were predominant motivational factors for sport participation [12]. A narrative review of studies involving children, adolescents and adults corroborated the findings [15]. However, our results also show that there are unique motives for rugby participation among schoolboys facilitated by contextual, environmental and rugby-related factors. Sports are compulsory in Zimbabwean schools and schoolchildren partake in rugby by choice regardless whether they are selected or not for competitive matches. In such contexts, participation is driven more by passion and love for the game despite the physical challenges inherent in the sport. Additionally, issues around masculinity and physicality were also expressed as motivating factors. This is linked to the nature of sport which requires a show of masculine ego and physical attributes commensurate with rugby demands [5–8]. Extrinsic motivational factors such as professional ambitions, monetary rewards were also identified. Similarly, they have been reported elsewhere [13, 31] and reflect contextual and socio-cultural perceptions people have towards the sport.

The study also explored coaches’ criteria when selecting schoolboys for inclusion in school rugby teams. Coaches largely select based on training performances, players’ attitudes, physical fitness, physical qualities, and possession of basic rugby skills. This variability indicates different coaching ideologies and guiding principles for team selection. These findings are supported by previous studies and reflect the nature of the sport which requires technical competence and well-developed physical attributes [23–25]. To achieve that expertise, training is required and this explains value placed on training performances by coaches; a finding shared by Baker et al. [32].

## Limitations

- This study did not interview schoolboy players. Future studies may want to triangulate these findings using them.
- Data analysis was not conducted iteratively based on the saturation concept. Therefore, it is not clear if saturation was reached with 22 participants included in the sample.

## Additional files

**Additional file 1.** Emergent codes, categories and themes from participatory behaviour data from interviewing high school adolescent rugby coaches.

**Additional file 2.** Emergent codes, sub-categories, categories and themes from the interview data on coaches’ selection criteria.

## Abbreviations

CESRL: Co-educational School Rugby League
DZSRF: Dairiboard Zimbabwe School Rugby Festival
IHSRL: Interscholastic School Rugby League
HREC: Human Research Ethics Committee
SESRL: Super Eight School Rugby League
SCRuM: School Clinical Rugby Measure
U: under

## References

[1] World Rugby. Playing numbers. https://www.world.rugby/development/player-numbers. Accessed 15 Nov 2018.

[2] Archibold HAP, Rankin AT, Webb M, Nicholas R, Earnes NWA, Wilson RK (2017). RISUS study: rugby injury surveillance in ulster schools. Br J Sports Med.

[3] Leung FT, Smith-Franettovich MM, Hides JA (2017). Injuries in Australian school-level rugby union. J Sports Sci.

[4] Chiwaridzo M, Masunzambwa Y, Naidoo N, Kaseke F, Dambi JM, Matare T (2015). Profile of rugby injuries in high school Zimbabwean adolescents. Int J Sports Exerc Sci.

[5] Durandt J, Green M, Masimla H, Lambert M (2018). Changes in body mass, stature and BMI in South Africa elite U18 Rugby players from different racial groups from 2002–2012. J Sports Sci.

[6] Lombard WP, Durandt JJ, Masimla H, Green M, Lambert M (2015). Changes in body size and physical characteristics of South African under-20 rugby union players over a 13-year period. J Strength Condition Res.

[7] Read DB, Jones B, Phibbs PJ, Roe GAB, Darrall-Jones J, Weakley JS (2018). The physical characteristics of match-play and academy rugby union. J Sports Sci.

[8] Read D, Weaving D, Phibbs P, Darral-Jones J, Roe G, Weakley JS (2017). Movement and physical demands of school and university rugby union match-play in England. BMJ Open Sport Exerc Med.

[9] Bleakley C, Tully M, O’Connor S (2011). Epidemiology of adolescent rugby injuries: a systematic review. J Athletic Train.

[10] Burger N, Lambert MI, Viljoen W, Brown JC, Readhead C, den Hollander S (2016). Mechanisms and factors associated with tackle-related injuries in South African youth rugby union players. Am J Sports Med.

[11] Chiwaridzo M, Chandahwa D, Oorschot S, Tadyanemhandu C, Dambi JM, Ferguson G (2018). Logical validation and evaluation of practical feasibility for the SCRuM (School Clinical Rugby Measure) test battery developed for young adolescent rugby players in a resource-constrained environment. PLoS ONE.

[12] Kilpatrick M, Herbert E, Bartholomew J (2010). College students motivation for physical activity: differentiation men’s and women’s motives for sports participation and exercise. J Am Coll Health.

[13] Crane J, Temple V (2015). A systematic review of dropout from organised sport among children and youth. Eur Phys Educ Rev.

[14] Chen C, Tsai LT, Lin CF, Huang CC, Chang YT, Chen RY (2017). Factors influencing interest in recreational sports participation and its rural-urban disparity. PLoS ONE.

[15] Allender S, Cowburn G, Foster C (2006). Understanding participation in sport and physical activity among children and adults: a review of qualitative studies. Health Educ Res.

[16] Bragaru M, Van Wilgen CP, Geertzen JHB, Ruijs SGJB, Dijkstra PU, Dekker R (2013). Barriers and facilitators of participation in sports: a qualitative study on dutch individuals with lower limb amputation. PLoS ONE.

[17] Bailey R, Cope EJ, Pearce G (2013). Why do children take part in, and remain involved in sport? A literature review and discussion of implications for sports coaches. Int J Coach Sci.

[18] Kondric M, Sindik J, Furjan-Mandic G, Schiefler B (2013). Participation motivation and student’s physical activity among sport students in three countries. J Sports Sci Med.

[19] Moreno JA, Gonzalez-Cutre D, Martin-Albo J, Cervello E (2010). Motivation and performance in physical education: an experimental test. J Sports Sci Med.

[20] Clancy RB, Herring MP, MacIntyre TE, Campbell MJ (2016). A review of competitive sport motivation research. Psychol Sport Exerc.

[21] Spray CM, Wand CKJ, Biddle SJH, Chatzisarantis NLD (2006). Understanding motivation in sport: an experimental test of achievement goal and self-determination theories. Eur J Sport Sci.

[22] Vallerand RJ, Losier GF (1999). An integrative analysis of intrinsic and extrinsic motivation in sport. J Appl Sport Psychol.

[23] Gabbett TJ, Jenkins DG, Abernethy B (2011). Relative importance of physiological, anthropometric, and skill qualities to team selection in professional rugby league. J Sports Sci.

[24] Gabbett T (2002). Influence of physiological characteristics on selection in a semi-professional first grade rugby league team: a case study. J Sports Sci.

[25] Gabbett TJ (2009). Physiological and anthropometric characteristics of starters and non-starters in junior rugby league players, aged 13-17 years. J Sports Med Phys Fitness.

[26] Chiwaridzo M, Munambah N, Oorschot S, Magume D, Dambi JM, Ferguson G, et al. Coaches perceptions on qualities defining good adolescent rugby players and are important for player recruitment in talent identification programs: the SCRuM project. BMC Research Notes. 2018. Under Review. 10.1186/s13104-019-4170-y PMC6417159 30867064

[27] Hsieh HF, Shannon SE (2005). Three approaches to qualitative content analysis. Qual Health Res.

[28] Bengtsson M (2016). How to plan and perform a qualitative study using content analysis. NursingPlus Open..

[29] Hollembeak J, Amorose AJ (2005). Perceived coaching behaviours and college athletes’ intrinsic motivation: a test of self-determination theory. J Appl Sport Psychol.

[30] Vlachopoulos SP, Karageorghis CI, Terry PC (2000). Motivation profiles in sport: a self-determination theory perspective. Res Q Exerc Sport.

[31] Neely KC, Holt NL (2014). Parents’ perspectives on the benefits of sport participation for young children. Sport Psychol.

[32] Baker J, Horton S, Robertson-Wilson J, Wall M (2003). Nurturing sport expertise: factors influencing the development of elite athlete. J Sports Sci Med.

