# Supplementary material for: High-school adolescents’ motivation to rugby participation and selection criteria for inclusion in school rugby teams: coaches’ perspective (the SCRuM project)
Source: BMC Res Notes. 2019 Feb 26;12:103. doi: 10.1186/s13104-019-4138-y (PMC6390623; doi:10.1186/s13104-019-4138-y)
Supplement: Supplementary file 1 — Additional file 1. Emergent codes, categories and themes from participatory behaviour data from interviewing high school adolescent rugby coaches. [file 13104_2019_4138_MOESM1_ESM.docx]

**Additional file 1:** Themes, categories, emergent codes for the participatory behaviour interview data

| **Theme** | **Category** | **Sub-category** | **Emergent codes** | **Condensed meaning units** | **Meaning units** |
| --- | --- | --- | --- | --- | --- |
| It’s a choice to play rugby | Intrinsic motivation | Personal preference | Rugby is optional | Rugby is open to every child | “The sport is open to every child, not just boys but even for girls these days. In all primary and secondary schools, any child who wants to play, we encourage them to join in and play. As a matter of fact, the Ministry, our own Ministry of Primary and Secondary Education in this country, mandates every child to participate in sport, in sport of their choice, as part of their education”(Participant AW01) |
| It’s a choice to play rugby | Intrinsic motivation | Personal preference | Rugby is optional | Rugby is a sport for everyone | “You know rugby is a sport for everyone, not just for the 30 players you see running around for the ball in the pitch, there are many students who play rugby who probably will not be fielded in any of these matches, or they have been left for various reasons. Its sport for every kid out there who want to experience rugby and who want to enjoy the sport” (Participant STG01) |
| It’s a choice to play rugby | Intrinsic motivation | Personal enjoyment | Rugby is optional | It’s a sport for every kid wanting to experience and enjoy the sport | “You know rugby is a sport for everyone, not just for the 30 players you see running around for the ball in the pitch, there are many students who play rugby who probably will not be fielded in any of these matches, or they have been left for various reasons. Its sport for every kid out there who want to experience rugby and who want to enjoy the sport” (Participant STG01) |
| It’s a choice to play rugby | Intrinsic motivation | Passion | Passion for the game | Youngsters are motivated by the passion for the game | “I think what motivates these youngsters to play rugby is passion for the game, the kids enjoy the sport and that is the biggest push factor. If you consider how dangerous the sport is, you can get bruised, you can get wrangled, you can get tackled down to the hard ground, look at your lawn less grounds, you can get pulled or pushed, you get a concussion and so forth and still you have 30, 40 kids coming and say they want to play rugby. That is passion” (Participant CBC01) |
| It’s a choice to play rugby | Intrinsic motivation | Personal enjoyment | Enjoy the game | The kids enjoy the game | “I think what motivates these youngsters to play rugby is passion for the game, the kids enjoy the sport and that is the biggest push factor. If you consider how dangerous the sport is, you can get bruised, you can get wrangled, you can get tackled down to the hard ground, look at your lawn less grounds, you can get pulled or pushed, you get a concussion and so forth and still you have 30, 40 kids coming and say they want to play rugby. That is passion” (Participant CBC01) |
| It’s a choice to play rugby | Intrinsic motivation | Passion | Passion for the game | It’s all I feed on everyday | “I remember asking one Under 16 rugby player, after he walked out of a competitive match last year, why he is still interested in playing rugby after having a fracture of collarbone. He said to me rugby is everything to me; it’s all I feed on every day. I read, sleep and talk rugby every time and you realise that such kids with passion, will go on and make it in rugby whether as adolescent rugby players or as academy players or senior professional rugby players because they do not allow anything to stand in their way even an injury to the collarbone could not stop him play. Today, he is back again, playing at the same pitch that injured him and still playing the same sport which fractured his bone. You can imagine” (Participant MF01) |
| It’s a choice to play rugby | Intrinsic motivation | Nature of sport | The adrenaline rush | Most students enjoy the adrenaline rush that comes with the sport | “Watch when my boys play school A, watch when my boys play School B, it’s like war, watch when School C play School D, you like it, you enjoy school rugby, and most students who come for rugby enjoy the adrenaline rush that comes with tough and competitive sports” (Participant AW01) |
| It’s a choice to play rugby | Intrinsic motivation | Love of the sport | Love for the sport | The kids love the sport | “I think the kids love the sport, and I would say which sport can accommodate players of different sizes of people like rugby. You see very fat kids play rugby, very thin kids playing rugby and in between players playing rugby, very strong and not so strong players playing rugby, so I think kids love it for that reason, it’s a sport that embraces everyone and accommodates different people of different sizes” (Participant LOM01) |
| It’s choice to play rugby | Intrinsic motivation | Nature of the sport | Accommodativeness of the sport | The sport accommodates players of different sizes | “I think the kids love the sport, and I would say which sport can accommodate players of different sizes of people like rugby. You see very fat kids play rugby, very thin kids playing rugby and in between players playing rugby, very strong and not so strong players playing rugby, so I think kids love it for that reason, it’s a sport that embraces everyone and accommodates different people of different sizes” (Participant LOM01) |
| It’s a choice to play rugby | Intrinsic motivation | Nature of the sport | Competitiveness of the sport | The excitement of playing competitive rugby | “First, at School E, we play elite rugby, we play competitive rugby and so that excitement of playing competitive rugby in a sport that attracts a huge crowd that school probably makes them want to play rugby for the school” (Participant F01). |
| It’s a choice to play rugby | Intrinsic motivation | Nature of the sport | Attracts a huge crowd | Excitement of playing competitive rugby in a sport that attracts a huge crowd | “First, at School E, we play elite rugby, we play competitive rugby and so that excitement of playing competitive rugby in a sport that attracts a huge crowd that school probably makes them want to play rugby for the school” (Participant F01). |
| It’s a choice to play rugby | Extrinsic motivation | Professional ambitions | Playing professional rugby | Most boys play with the hope of playing professional rugby | “So, most of these boys who play rugby, play rugby with the hope and the notion that they will play professional rugby at some point in their life at a distant future and probably make a lot of money. That mentality drives their passion, feeds their love of the game and they feel motivated to want to play rugby “ (Participant MF01) |
| It’s a choice to play rugby | Extrinsic motivation | Emulation | Emulation | We have coach X who came through junior ranks and played for country | “We have the likes of X, who came through the junior ranks, played for the national team, played outside Zimbabwe and now they are back coaching these kids. That is strong motivation for the kids to want to play rugby for the school” (Participant PE01) |
| It’s a choice to play rugby | Extrinsic motivation | Peer Influence | Peer influence | Some are dragged by others | “Some are dragged by others, especially if you look at the U13s, most of them will drop the sport by the time they get to form three or four. They will realise that, this is a challenging sport and it’s not for me or maybe their parents will not advise them to play and they will quit” (Participant ES01) |
| It’s a choice to play rugby | Extrinsic motivation | Parental influence | Parental influence | Other influences from parents | “They are also other influences such as parental support and pressure to play rugby, maybe from parents who used to play the sport when they were young, or used to enjoy watching the sport or are still watching the school fervently. It happens a lot, the father is a soccer coach, and the kid wants to play soccer” (Participant AW01) |
