# Supplementary material for: High-school adolescents’ motivation to rugby participation and selection criteria for inclusion in school rugby teams: coaches’ perspective (the SCRuM project)
Source: BMC Res Notes. 2019 Feb 26;12:103. doi: 10.1186/s13104-019-4138-y (PMC6390623; doi:10.1186/s13104-019-4138-y)
Supplement: Supplementary file 2 — Additional file 2. Emergent codes, sub-categories, categories and themes from the interview data on coaches’ selection criteria. [file 13104_2019_4138_MOESM2_ESM.docx]

**Additional file 2**: Themes, categories, meaning units for coaches’ selection criteria interview data

| **Theme** | **Category** | **Emergent codes** | **Condensed meaning unit** | **Meaning unit** |
| --- | --- | --- | --- | --- |
| It is about the player | Training performance | Brilliant in training | Youngsters have to be brilliant in training | *“The youngsters that have been brilliant in training, working very hard during training as seen by the coach probably get a nod to play. We all have to agree on which players to play” (Participant PE02)* |
| It is about the player | Attitude | Positive attitude | I choose players that are hardworking, motivated principled with the commitment and discipline | *“For example as the head coach for the U13, I choose players that are hardworking, these are youngsters and rugby is a principled sport so we expect the boys to have principles, to show that they are committed, motivated to play and have discipline, it’s difficult to deal with form one because they think rugby is just running and tackling so we tend to emphasise on team work, willing to learn and i tend to choose players that are show desire to work with the coach and improve their skills” (Participant ER01)* |
| It is about the player | Attitude | Positive attitude | I emphasise on team work, willing to learn and show of desire to work with coach and improve skills | *“For example as the head coach for the U13, I choose players that are hardworking, these are youngsters and rugby is a principled sport so we expect the boys to have principles, to show that they are committed and have discipline, it’s difficult to deal with form one because they think rugby is just running and tackling so we tend to emphasise on team work, willing to learn and i tend to choose players that are show desire to work with the coach and improve their skills” (Participant ER01)* |
| It is about the player | Physical qualities | Physical qualities | They have to have the physical qualities important in rugby | *“But of course they have to have the physical qualities and the skills that important in rugby, and they have to be injury free. All the players we have in our team were chosen out of hundreds to students who wanted to play for the first team so we don’t doubt their capabilities, their skills”(Participant P01)* |
| It is about the player | Skills | Skills | They have to have the skills | *“But of course they have to have the physical qualities and the skills that important in rugby, and they have to be injury free. All the players we have in our team were chosen out of hundreds to students who wanted to play for the first team so we don’t doubt their capabilities, their skills” (Participant P01)* |
| It is about the player | Physical qualities | Physical qualities | The sport is for strong and we expect all players to strength and balance | *The sport is for the strong whether you are playing as a winger, flank, scrum half and fly half there is a degree of physical fitness that we expect all rugby players to have, minimum physical fitness, strength in your trunk, legs, arms, neck and good balance such that if you are pushed you will fall down like a sack. At least you can withstand the massive forces that come with tackles, and so forth. So for me, I usually consider physique a criteria for choosing players” (Participant MF01)* |
| It is about the player | Rugby skills | Skills | We pick players who can run, pass, strong to tackle | *“…but certainly we would pick players who can run, who can hold the ball, pass and score, and players with the commitment to play, to play to win, players who are strong to tackle, when tackled, players who can fight to win possession of the ball and so forth” (Participant ES02)* |
| It is about the match | Balanced team | Balanced team | We go for players who give a balanced team | *“I try to go for players who give me a balanced team, players with strength and power up front, with very good tackling proficiency and very good passing and catching ability, and then have also defenders, or backline players with a keen sense of the game, who can read the game, with vision, speed and catching and passing abilities, as well as kicking. So the team must be balanced, strong in front but very fast on the back. So when we select players we try to get players who can achieve that balance for us.”* |
| It is about the match | Opponent team | Opponent team | Have good knowledge of your opponents | *“I can tell if you bring a team from the super eight and play it with a team from the interscholastic amateur league, it will be a whitewash. So as a coach, you need to have good knowledge of your opponents, their strength and weakness and form strategise and see which players from your team you need to include to make up the team” (Participant STG01)* |
